# Structure–Property Relationship of Polymerized Ionic Liquids for Solid-State Electrolyte Membranes

**DOI:** 10.3390/polym13050792

**Published:** 2021-03-04

**Authors:** Robert Löwe, Thomas Hanemann, Tatiana Zinkevich, Andreas Hofmann

**Affiliations:** 1 Institute for Applied Materials, Karlsruhe Institute of Technology, Hermann-von-Helmholtz-Platz 1, D-76344 Eggenstein-Leopoldshafen, Germany; robert.loewe@kit.edu (R.L.); thomas.hanemann@kit.edu (T.H.); tatiana.zinkevich@kit.edu (T.Z.); 2 Department of Microsystems Engineering, University of Freiburg, Georges-Köhler-Allee 102, D-79110 Freiburg, Germany

**Keywords:** polymerizable ionic liquids, ionogel, structure–property relationship, ionic conductivity, ionic liquids, lithium-ion battery, solid state electrolyte, solid-state-battery

## Abstract

Eight new polymerized ammonium-based ionic liquids were prepared as thin membrane films and evaluated within the scope of their usage in lithium-ion batteries. The focus of this work is to get a better understanding of the influence of structural modifications of the monomers on the polymerized materials. Further, different concentrations of a lithium-ion conducting salt were applied in order to receive an optimized combination of monomer structure and lithium salt concentration. It was found that an increased side chain length of the studied ammonium-based polymerized ionic liquids leads to a reduction in glass transition temperatures and increased ionic conductivity values. As a result of the addition of conducting salt to the PIL membranes, the glass transition temperatures and the ionic conductivity values decreases. Nevertheless, PFG-NMR reveals a higher lithium-ion mobility for a sample with higher conducting salt content.

## 1. Introduction

In terms of battery electrolytes, ionic liquids feature several desirable properties like low vapor pressure, low flammability, high ionic conductivity and high electrochemical stability. [1] Thus, transferring these properties into a solid electrolyte system would be promising for increasing safety of lithium-ion batteries. One approach is the covalent linkage of ionic liquid molecules via direct polymerization of an acrylate or methacrylate function. The resulting material class of polymerized ionic liquids (PIL) was pioneered by the group Ohno et al. [2,3,4] and meanwhile evaluated for several different applications alongside the battery research, like in solar cells [5] or for CO_2_ adsorption [6]. Compared to conventional separator-liquid electrolyte systems, all-solid-state polymer electrolytes are expected to be beneficial in terms of battery safety due to properties like no leakage or high thermal stability [7]. The most widely investigated material class for this type of application are poly(ethylene oxide) (PEO) based membranes [8]. The huge drawback of PEO based solid electrolytes is the low ionic conductivity at room temperature due to glass transition temperatures far above room temperature and resulting slow segmental movements of the polymer chains [8]. The approach of combining the ionic liquids physical properties in a solid material with an almost uncountable variety of combinations with different anions and variations in the cations structure enables an outstanding tunability of the materials properties. To overcome challenging issues as low ionic conductivity values but also to get a better understanding of systematical coherences within the variation possibilities, systematical structure–property studies are necessary. For the usage of PIL materials with a cationic polymer backbone as an alternative to conventional separator-electrolyte systems, at least two components are necessary, (i) the PIL structure and (ii) an lithium-ion conducting salt within the polymer matrix.

Shaplov et al. define three parts of the structure building ionic liquid monomers, (i) the polymerizable function, (ii) the spacer and (iii) the ionic group [9]. Properties of the resulting polymer can be adjusted by variations in each of these parts, plus variation of the counter ion species. There is a large amount of literature covering different variations and resulting material properties. Although there is a huge variety of studied structures, there is a lack of systematic research in structure–property relationships, especially when it comes to the combination of the polymers structure and the influence of the conducting salt concentration.

**Figure 1:**
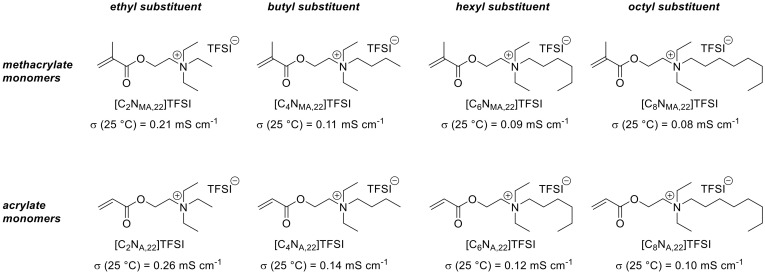
Previously introduced ionic liquid monomers and their ionic conductivities at 25 °C [10].

In order to get an impression of how the monomers structure influences the polymers properties, we synthesized eight new ammonium-based ionic liquid monomers, which were presented in a previous publication [10]. Figure 1 shows the corresponding chemical structures which are labelled as [C_n_N_(M)A,22_]TFSI. Variations were applied in the polymerizable group and the length of one alkyl group at the quaternary ammonium group. For all monomeric ionic liquids the Bis(trifluoromethane)sulfonimide anion (TFSI) was used. C_n_ represents the length of the introduced alkyl chain at the quaternary ammonium ion. The synthesis of the monomers starts either from 2-(diethylamino)ethyl acrylate or 2-(diethylamino)ethyl methacrylate, which is indicated in the nomenclature as A,22 (for acrylate and two ethyl groups) or MA,22 (for methacrylate and two ethyl groups), respectively. For the monomers (labelled as [Cation]Anion), we found that the structure variations have a systematic influence on the monomer’s ionic conductivity. Acrylate functionalities and short side chains at the ammonium group lead to higher ionic conductivities than methacrylates and longer side chains. This observation can be explained with the higher steric demand of bulkier groups and resulting phenomena like entanglements that lead to increased internal friction, causing decreased ionic mobility [10].

In this manuscript, we will discuss how the monomers structure influences the polymer properties. Further, different concentrations of LiTFSI as a conducting salt were added to the polymer matrix to build lithium-ion conducting membranes. The results will be discussed in terms of an application as solid electrolyte membranes for lithium-ion batteries.

## 2. Materials and Methods

### 2.1. Materials

The polymerizable ionic liquids [C_2_N_MA,22_]TFSI, [C_4_N_MA,22_]TFSI, [C_6_N_MA,22_]TFSI, [C_8_N_MA,22_]TFSI, [C_2_N_A,22_]TFSI, [C_4_N_A,22_]TFSI, [C_6_N_A,22_]TFSI and [C_8_N_A,22_]TFSI were synthesized as described in our previous publication [10]. Bis(trifluoromethane)sulfonimide lithium salt (99.95% on metal basis) and the initiator Irgacure651 were purchased from Sigma-Aldrich (Munich, Germany) and Ciba Chemicals (Basel, Switzerland) and used as received. For FT-NMR spectroscopy, polymer samples were dissolved in acetone-d_6_ (99.80%; Deutero, Germany).

### 2.2. Polymerisation Equipment

Polymerization of the IL monomers was carried out in an *EC-500* UV chamber (Electro-lite, Bethel, CT, USA), equipped with four 9 W UV lamps peaking at 365 nm (P/N 82469; Electro-lite, Bethel, CT, USA). The UV-chamber was located inside an argon-filled glovebox (H_2_O, O_2_ < 0.5 ppm) to prevent water contamination.

### 2.3. Measurements

NMR spectra were recorded using an *Avance III HD 500 MHz* spectrometer (Bruker, Rheinstetten Germany). Glass transition temperatures (accuracy ± 2.5 °C) were measured by DSC analysis using a *DSC 204 F1 Phoenix* (Netzsch, Selb, Germany) with temperature programs between −150 °C and 180 °C with heating rates of 10 K min^−1^. The given glass transition temperatures are the inflection points of the heating curves and were calculated with support of the software *Proteus* (Netzsch, Selb, Germany). DSC coupled TG analysis (TGA) was carried out on a *STA 449F3 Jupiter* apparatus (Netzsch, Selb, Germany) with synthetic air (80/20) as purge gas (250 mL·min^−1^).

Ionic conductivity values were received from electrochemical impedance spectroscopy. For the measurements, a *Zahner Zennium* potentiostat (Zahner, Kronach, Germany) and Swagelok type cells (Swagelok, Stuttgart, Germany) located inside a *SH-261* climate chamber (ESPEC, Düsseldorf, Germany) were applied. The spectra were recorded between 2 MHz and 10 mHz with an amplitude of 10 mV. Within the Swagelok cells, the PIL membranes were located between two non-lithium containing blocking electrodes. The membranes bulk resistance R was determined at the point where the phase difference approaches 0°. The resulting ionic conductivity values *σ* are calculated from the bulk resistance values *R*, the electrodes surface area *A* and the PIL membranes thickness *l* via the equation 1 [11]:

(1) $$\sigma =\frac{1}{R}\cdot \frac{l}{A}$$

Both, the surface area A and the PIL membranes thickness l are determined individually for every single experiment directly after the EIS experiment was executed. Typical electrode diameters were 12.3 to 12.5 mm and measured with a sliding caliper. The thickness of the PIL membranes was quantified with a *VRZ 4* measuring sensor (Heidenhain, Germany) and was typically between 60 μm and 150 μm.

Self-diffusion coefficient measurements were performed on a Bruker NMR spectrometer, which operates at the ^7^Li resonance frequency of 116.6 MHz. The spectrometer was equipped with a pulse-field gradient (PFG) probehead that produces linear z-gradients with strengths up to 0.3 T·cm^−1^. Stimulated echo (STE) pulse sequence with bipolar gradients allows to avoid issues with fast *T_2_*-relaxation in solids and the eddy currents appearing when one deals with strong gradients [12]. The diffusion parameters were optimised and reached 3 ms for the gradient duration (*δ*) and 100 ms for the diffusion delay (Δ). In the experiment, the signal intensity is measured as a function of the gradient strength (*g*) and this obeys Stejskal-Tanner equation: [13]

(2) $$I\left(t\right)\sim I_{0}\Delta exp\left(-\frac{\Delta }{T_{1}}\right)\Delta \exp\left(-D\gamma^{2}g^{2}\delta^{2}\left(\Delta -\frac{\delta }{3}\right)\right)$$

where *I*_0_ is the initial magnetization, *T*_1_ denotes the spin-lattice relaxation time, γ is the gyromagnetic ratio of the ^7^Li nucleus. In order to get rid of the relaxation time dependence, all timings were kept constant through the whole pulse sequence.

## 3. Results and Discussion

### 3.1. Preparation of the Polymer Films

**Figure 2:**
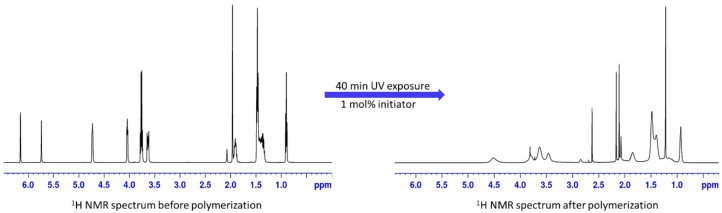
^1^H NMR spectrum of [C_2_N_MA,22_]TFSI before and after polymerization. Samples were dissolved in acetone-d_6_.

Due to the usage of hygroscopic materials, preparation of the PIL membranes (labelled as P[Cation]Anion) was performed inside an argon-filled glovebox. Ionic liquid monomers were mixed with the claimed amount of conducting salt LiTFSI and 2,2-Dimethoxy-2-phenylacetophenone as a photo initiator for radical polymerization. The initiator concentration was set to 0.01 mol initiator per 1 mol ionic liquid monomer. The freshly prepared monomer mixtures were applied on an aluminum foil with a doctor’s blade (200 μm gap) applicator and cured inside an UV oven. It was found, that the liquid monomer mixtures turn rapidly into solid materials after a few seconds of UV exposure. Nevertheless, the sheets were treated 40 min with UV-radiation (365 nm) to ensure a high conversion rate of the (meth-) acrylate groups. All polymers were manufactured with the same preparation method. Figure 2 shows exemplarily the ^1^H NMR spectrum of [C_2_N_MA,22_]TFSI before and after the polymerization. The typical thickness of the prepared membrane films is between 60 μm and 150 μm.

As seen in the NMR spectra, the two proton signals at the double bond at 5.74 ppm and 6.13 ppm chemical shift disappear completely during the polymerization. In terms of detection levels of NMR spectroscopy, the polymerization occurs quantitatively. New proton signals in the polymer sample occur at 1.22 ppm and between 2 ppm and 3 ppm and derive from the aliphatic protons in the polymer backbone and the aliphatic side chain proton in α-position to the backbone.

### 3.2. Structure–Property Relationship of the Polymerized Ionic Liquids

**Figure 3:**
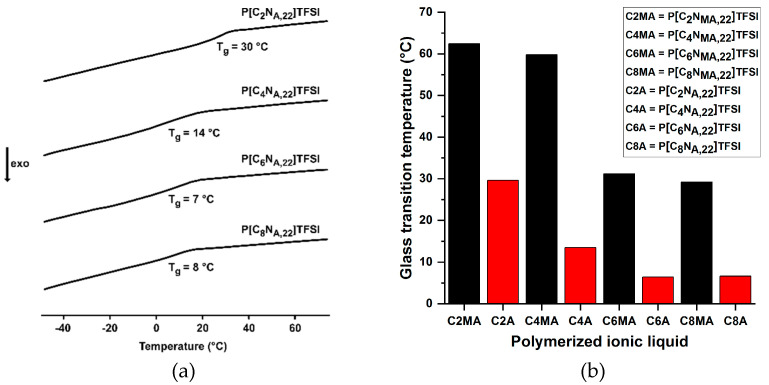
(**a**) Exemplary DSC data for the neat acrylate-PIL materials (**b**) Glass transition temperatures of the polymerized methacrylate (MA) and acrylate (A) containing ionic liquids.

It has been observed that the various polymerized ionic liquids show significant differences in their hardness and brittleness. The methacrylate PIL are more brittle and harder than the acrylate IL. Furthermore, longer side chains on the ammonium ion lead to softer polymers. At room temperature, P[C_2_N_MA, 22_]TFSI is a hard, brittle glass, whereas P[C_8_N_A, 22_]TFSI is a relatively soft, viscoelastic material. The different mechanical behavior can be derived from the glass transition temperatures T_g_ determined by means of differential scanning calorimetry (DSC), which are graphically summarized in Figure 3.

The glass transition temperatures of the polymers tend to decreases with increasing chain length of the substituted alkyl chain. The acrylate derivatives have significantly lower glass transition temperatures than the corresponding methacrylates at the same side chain length. The influence of the alkyl chain length on T_g_ is particularly pronounced between the ethyl- and hexyl-substituted IL. The hexyl- and octyl-derivatives of the respective homologous series differ only slightly in their T_g_ values. From Figure 3b it can also be seen, that the methacrylate PIL materials occur in a glassy state at room temperature. Thus, they show a much higher rigidity than the acrylates at room temperature.

**Figure 4:**
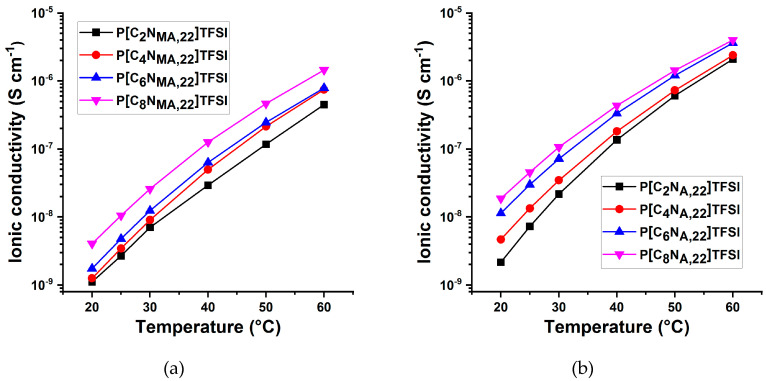
Ionic conductivities of the neat (**a**) methacrylate PIL (**b**) acrylate PIL membranes at various temperatures.

Ionic conductivities were determined in Swagelok type cells against nickel as a blocking electrode via electrochemical impedance spectroscopy (EIS). Figure 4 shows the ionic conductivities of all eight neat PIL systems at various temperatures. As expected, the ionic conductivities of all PIL increase with increasing temperature. As found for the monomers, the acrylates feature higher ionic conductivities than the methacrylate derivatives. The analysis also reveals an opposite influence of the side chain length on the ionic conductivity compared to the monomers. With increasing side chain length, the ionic conductivities increase. Zhang et al. synthesized and analyzed the two similar methacrylate PIL P[C_4_N_MA,11_][TFSI] and P[C_7_N_MA,11_][TFSI] with two methyl units and a butyl or heptyl substituent at the cationic center, respectively [14]. The P[C_4_N_MA,11_][TFSI] with the shorter side chain has a glass transition temperature of 66 °C and a ionic conductivity of approximately 2·10^−9^ S·cm^−1^ [14]. The heptyl derivate P[C_7_N_MA,11_][TFSI] features a lower glass transition temperature of 48 °C and a higher ionic conductivity of around 1·10^−8^ S·cm^−1^ [14]. This is consistent with the systematic influence of the side chain length we found and shows that this structure–property relationship may also apply for similar TFSI-PIL.

The structure–property trends for increasing ionic conductivity values correlate strongly with the decreasing glass transition temperatures. This indicates that the segmental mobility has a strong influence on the ionic conductivities of the PIL. As the literature shows, the same trend was also found for several other PIL materials [14,15,16]. It is assumed, that the ionic conductivity within the neat PIL is almost exclusively caused by the TFSI anions, since the cations are bound within in the polymer matrix. Larger side chains cause a higher steric demand and a higher free volume, what is proven by decreasing glass transition temperatures. Besides the influence of the segmental movement, the higher ionic conductivity may be explained by two more effects, i) more free space for the anions to move between the polymer chains and ii) a higher electrochemical shielding of the cationic groups in the backbone resulting in less electrostatic attraction between the cations and TFSI anions. As discussed in the literature, electrochemical shielding of the positively charged nitrogen center due to longer alkyl chains may influence properties like the cationic stability of an ionic liquid [17]. Therefore, it is assumed, that this kind of electrochemical shielding may also affect the electrostatic interactions between the positively charged ammonium center and TFSI anions within a polymerized ionic liquid.

**Figure 5:**
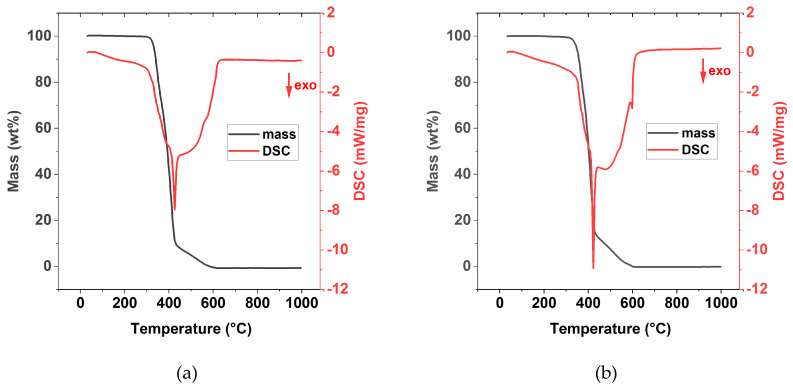
Graphical representation of the results of the TGA-DSC analysis of (**a**) P[C_2_N_MA,22_]TFSI; (**b**) P[C_2_N_A,22_]TFSI.

Coupled thermogravimetric analysis with DSC (TGA-DSC) measurement shown in Figure 5 reveal that the decomposition of the polymers are exothermic reactions. TGA curves of all eight neat PIL materials are provided in the Supplementary Materials Figure S1. The DSC analysis also shows that there is no melting peak between 25 °C and 300 °C. It can therefore be assumed that the polymers have a predominant amorphous structure. All eight neat PIL materials feature very high thermal stabilities with decomposition temperatures above 300 °C.

Table 1 summarizes the ionic conductivity values of all neat PIL at room temperature and 60 °C, glass transition temperatures and thermostability values. The data show that T_g_ may have a pronounced impact on the ionic conductivity at room temperature. Increasing of the temperature leads to a less marked increasing of ionic conductivity for samples with T_g_ below room temperature compared to samples with T_g_ above 25 °C. The membranes of disassembled cells with P[C_8_N_A,22_]TFSI after EIS measurements showed that the PIL material was partly pressed out of gap between the electrodes. Thus, the acrylates with a long alkyl side chain might not be an appropriate choice for electrolyte membranes due to a low mechanical stability at higher temperatures, although they show the highest ionic conductivity values and lowest glass transition temperatures.

### 3.3. Influence of the Conducting Salt

**Figure 6:**
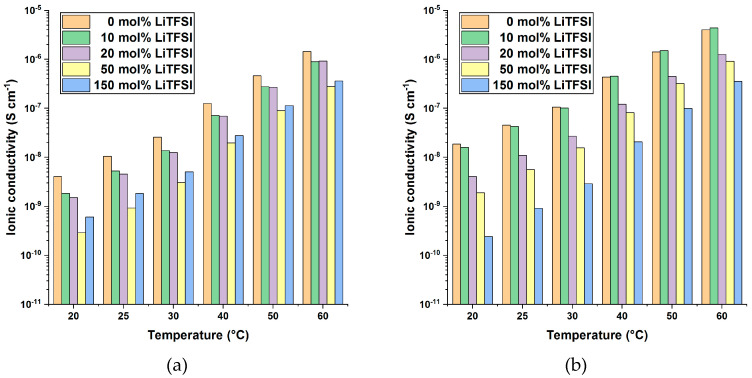
Ionic conductivity values of (**a**) P[C_8_N_MA,22_]TFSI and (**b**) P[C_8_N_A,22_]TFSI polymer membranes in dependence of temperature and LiTFSI concentration.

In order to study the influence of the conducting salt concentration on the membranes, LiTFSI was added to the monomers and the mixtures were polymerized like described for the neat PIL. The amount of LiTFSI was calculated in mol% which means [mol (LiTFSI) per mol (monomer)] to compensate varying molar masses of the different monomers. LiTFSI concentrations of 10, 20, 50 and 150 mol% were applied. By way of example, Figure 6 shows the resulting ionic conductivity values of P[C_8_N_A,22_]TFSI and P[C_8_N_MA,22_]TFSI in dependence of temperature and LiTFSI concentration (plots for all PIL membranes are available in the Supporting Materials Figure S2). Ionic conductivity values increases with increasing temperature and length of the alkyl side chain. With increasing content of conducting salt, the glass transition temperatures decreases as shown in Table 2 and the polymer membranes become noticeable softer, finally resulting in highly viscous fluid like materials for P[C_6_N_A,22_]TFSI and P[C_8_N_A,22_]TFSI. Thus, ionic density increases and T_g_ decreases, the ionic conductivity values overall tend to decrease due to the addition of conducting salt. Within the neat polymer membrane, the TFSI anions are the only moving ionic species since the cations are bound within the covalent network. Therefore, the ionic conductivity relates mainly to the anions. The data imply that an addition of lithium-ions to the matrix lowers not only the overall ionic conductivity but also the mobility of the TFSI anions. The observation of decreasing ionic conductivity values with increasing LiTFSI content might therefore be explained with a complex formation of Li cations and TFSI anions, reducing the anions mobility and therefore reducing the overall ionic mobility.

Table 3 shows the ionic conductivity values of P[C_2_N_A,22_]TFSI with varying LiTFSI content and compares it with literature data of two polyethylene oxide (PEO; M_W_ = 4 × 10^6^ g·mol^−1^) membranes containing different amounts of LiTFSI. The indexed number *n* in PEO_n_TFSI expresses the ratio of *n* ethylene oxide units to one lithium-ion (*n* EO:1 Li) [18]. The data show significant lower ionic conductivity values of the prepared PIL membranes compared to the literature values of the PEO membranes. Within the PEO matrix, lithium-ions are supposed to be coordinated by the oxygen atoms of the ethylene oxide (EO) units, which have a high flexibility, supporting the lithium-ion transport [19,20]. Transport of the lithium-ions is considered to proceed by ion hopping alongside the EO units via intrachain and interchain transfers [21,22]. The molecular structure of the studied PIL with cationic polymer backbone excludes these oxygen-related ion-hopping mechanisms. Instead, TFSI related transport mechanisms of the lithium-ions are proposed in the literature [23]. Even through, it is not possible to distinguish between the anions and cations influence on the ionic conductivity in the performed EIS measurements, comparable PEO membranes appear to have superior overall ionic conductivities.

The measurements of the ionic conductivity values by EIS give an easy accessible impression on the system. In terms of an application of PIL membranes in lithium-ion batteries, the mobility of lithium-ions is the more interesting property, but much more resource intense to access. To get an understanding of how the increasing lithium-ion salt concentration influences the actual lithium-ion mobility, PFG-NMR measurements were carried out on two samples. In our studies we found that P[C_2_N_A,22_]TFSI might be the most promising compromise of high ionic conductivity and mechanical stability. Thus, samples of P[C_2_N_A,22_]TFSI with 20 mol% and 150 mol% LiTFSI salt were used. As shown in Table 4, the diffusion coefficient of the sample with 20 mol% conducting salt is below the detection limit of 10^−14^ m^2^·s^−1^ at room temperature. Even though, the ionic conductivity of the sample with 150 mol% LiTFSI is almost six times lower, the diffusion coefficient of lithium is significantly higher. In view of an application as lithium-ion conducting membrane, the material with higher salt content has a superior lithium conducting property but unfortunately lower mechanical stability. In contrast to the neat PIL membranes, the addition of LiTFSI leads to a decoupling of the ionic conductivity from the segmental movement of the polymer chains. On the one hand, an addition of salt decreases the anion dominated ionic conductivity and increases the lithium-ion mobility on the other hand. This indicates that the transport of TFSI anions and lithium cations follow two different dominating mechanisms. While the lithium-ion mobility correlates with the segmental movement of the polymer chains, the anion mobility does not.

According to Zhang et al. the movement of lithium-ions through a TFSI-PIL system is coupled to a coordination of the lithium cations to the TFSI anions in different simultaneously occurring transport mechanisms [23]. The lithium-ions can either move as a kind of naked cations from one TFSI-coordination shell to another (structural movement) or within a fixed TFSI-complex shell from one cationic PIL center to another (vehicular movement) [23]. With increasing lithium concentration the overall lithium mobility increases and the vehicular movement is getting more influence over the structural movement [23]. Within the studied systems, the ionic conductivity is most likely dominated by the TFSI anions, since there is a significant excess of anions within the free moving ions. On the one hand, a higher anion density may lead to phenomena like electrostatic repulsion and therefore lead to lower ionic conductivity values in EIS. On the other hand, a higher LiTFSI content shifts the Li:TFSI ratio into direction of the lithium-ions, which may lead to a transport promoting coordination environment for the lithium-ions [23]. Therefore, a lower overall ionic conductivity but higher lithium mobility does not have to exclude each other.

## 4. Conclusions

In terms of a structure–property relationship for the PIL materials, the results reveal the following trends:

- Acrylate PILs feature lower T_g_ and higher ionic conductivity values than methacrylate PILs with same side chain length;
- Longer side chains lead to reduced T_g_ and increasing ionic conductivity values (within the studied range from C_2_ to C_8_ alkyl chains);
- A higher content of conducting salt reduces T_g_ and the ionic conductivity but increases the ^7^Li diffusion coefficient.

It was found that the structure of the ammonium-based IL monomers has a significant influence on the PIL membranes. Thereby, an opposite trend concerning the ionic conductivity was found for the neat PIL compared to the monomers. Increasing side chain lengths result in lower glass transition temperatures and higher ionic conductivity values, which is consistent with literature data found for P[C_4_N_MA,11_]TFSI and P[C_7_N_MA,11_]TFSI [14]. Methacrylates show lower ionic conductivity values than the acrylate derivates with same side chain lengths for both, the PIL and the monomers.

The addition of the lithium conducting salt LiTFSI leads in most cases to a decrease in the ionic conductivity values. Though, PFG-NMR studies revealed a higher lithium diffusion coefficient for a membrane with a higher LiTFSI concentration. However, for all tested membranes, the ionic conductivity values are significantly below 10^−4^ S·cm^−1^ at room temperature, which is one of the main requirements for the usage in lithium-ion batteries [24]. Furthermore, the influence of the structure modifications and conducting salt concentrations on the PIL membranes is significant, but it seems unlikely to reach a desirable electrochemical conductivity by adjustments of these two parameters for the studied ammonium-based PIL. Hence, for the usage of the studied PIL materials in battery cells, the addition of further additives is necessary to adjust electrochemical and mechanical properties. Nevertheless, the high thermal stability and very fast and easy processing to thin membrane layers makes PIL materials interesting candidates for polymer electrolyte membranes. Another big advantage is the combination of the high chemical stability of ionic liquids, brought together with the possibility to easily use other conducting salts. Accordingly, the PIL membranes are generally not just limited by the explicit usage for LiTFSI but may also be interesting for post lithium-ion cell systems such as lithium-sulfur or multivalent rechargeable batteries, or even in the field of [25].

## Figures and Tables

**Table 1:** Physicochemical properties of the neat polymerized ionic liquids, including ionic conductivity values at 25 °C, glass transition temperatures (T_g_), onset temperature of thermal decomposition (T_Dec_ (Onset)) and temperature at which 98 wt% of the sample mass is remaining in thermogravimetric analysis (T_Dec_ (98 wt%)).

Polymer	σ [S·cm^−1^] at 25 °C	σ [S·cm^−1^] at 60 °C	T_g_ [°C]	T_Dec_ (Onset) [°C]	T_Dec_ (98 wt%) [°C]
P[C_2_N_MA,22_]TFSI	2.7 × 10^−^^9^	4.5 × 10^−^^7^	62	333	323
P[C_4_N_MA,22_]TFSI	3.4 × 10^−^^9^	7.5 × 10^−^^7^	60	329	325
P[C_6_N_MA,22_]TFSI	4.8 × 10^−^^9^	7.9 × 10^−^^7^	31	330	329
P[C_8_N_MA,22_]TFSI	1.1 × 10^−^^8^	1.5 × 10^−^^6^	29	335	332
P[C_2_N_A,22_]TFSI	7.3 × 10^−^^9^	2.1 × 10^−^^6^	30	347	333
P[C_4_N_A,22_]TFSI	1.3 × 10^−^^8^	2.4 × 10^−^^6^	14	354	319
P[C_6_N_A,22_]TFSI	3.0 × 10^−^^8^	3.6 × 10^−^^6^	6	361	334
P[C_8_N_A,22_]TFSI	4.5 × 10^−^^8^	4.0 × 10^−^^6^	7	358	342

**Table 2:** Glass transition temperatures of the studied PIL-LiTFSI mixtures.

PIL	Glass Transition Temperature *T*_g_ of PIL Mixture with LiTFSI Content of				
	0 mol%	10 mol%	20 mol%	50 mol%	150 mol%
P[C_2_N_MA,22_]TFSI	62	31	29	20	−27
P[C_4_N_MA,22_]TFSI	60	14	8	7	−27
P[C_6_N_MA,22_]TFSI	31	3	−13	6	−43
P[C_8_N_MA,22_]TFSI	29	−3	−2	−2	−24
P[C_2_N_A,22_]TFSI	30	17	11	−7	−30
P[C_4_N_A,22_]TFSI	14	6	−1	−13	−35
P[C_6_N_A,22_]TFSI	6	6	0	−8	−40
P[C_8_N_A,22_]TFSI	7	0	−1	−11	−35

**Table 3:** Ionic conductivity values of P[C_2_N_A,22_]TFSI with varying LiTFSI concentrations compared to literature results for PEO-LiTFSI membranes. Literature values taken from [18].

Polymer	σ [S·cm^−1^] at 20 °C	σ [S·cm^−1^] at 60 °C
P[C_2_N_A,22_]TFSI (neat)	2.2 × 10^−^^9^	2.1 ×10^−^^6^
P[C_2_N_A,22_]TFSI + 10 mol% LiTFSI	2.4 × 10^−^^9^	2.7 × 10^−^^6^
P[C_2_N_A,22_]TFSI + 20 mol% LiTFSI	1.8 × 10^−^^9^	2.3 × 10^−^^6^
P[C_2_N_A,22_]TFSI + 50 mol% LiTFSI	1.8 × 10^−10^	7.9 × 10^−7^
P[C_2_N_A,22_]TFSI + 150 mol% LiTFSI	2.6 × 10^−10^	7.0 × 10^−7^
PEO_10_LiTFSI [18]	1.5 × 10^−^^5^	4.1 × 10^−4^
PEO_20_LiTFSI [18]	7.0 × 10^−^^6^	3.7 × 10^−4^

**Table 4:** Ionic condutivity values and diffusion coefficient of P[C_2_N_A,22_]TFSI with 20 mol% and 150 mol% LiTFSI at 25 °C.

Membrane	Ionic Conductivity at 25 °C [S·cm^−1^]C	^7^Li Diffusion Coefficient at 25 °C [m^2^·s^−1^]
P[C_2_N_A,22_]TFSI + 20 mol% LiTFSI	6.9 × 10^−9^	<10^−14^ *
P[C_2_N_A,22_]TFSI + 150 mol% LiTFSI	1.2 × 10^−9^	4.3 ± 0.1 × 10^−14^

* ^7^Li diffusion coefficient below detection limit of 10^−14^ m^2^·s^−1^.

## Supplementary Materials

The following are available online at https://www.mdpi.com/2073-4360/13/5/792/s1, Figure S1. TGA curves of the eight studied PIL materials without addition of conducting salt, Figure S2. Ionic conductivity values of the membranes in dependence of monomer structure, temperature and LiTFSI concentration.

## References

[1] Le Bideau J., Viau L., Vioux A. (2011). Ionogels, ionic liquid based hybrid materials. Chem. Soc. Rev..

[2] Ohno H. (2006). Functional Design of Ionic Liquids. Bull. Chem. Soc. Jpn..

[3] Ohno H. (2001). Molten salt type polymer electrolytes. Electrochimica Acta.

[4] Ohno H., Ito K. (1998). Room-Temperature Molten Salt Polymers as a Matrix for Fast Ion Conduction. Chem. Lett..

[5] Tseng S.-K., Wang R.-H., Wu J.-L., Jyothibasu J.P., Wang T.-L., Chu C.-Y., Lee R.-H. (2020). Synthesis of a series of novel imidazolium-containing ionic liquid copolymers for dye-sensitized solar cells. Polym..

[6] Tang J., Sun W., Tang H., Radosz M., Shen Y. (2005). Enhanced CO_2_ Absorption of Poly(ionic liquid)s. Macromolecules.

[7] Wang A., Liu X., Wang S., Chen J., Xu H., Xing Q., Zhang L. (2018). Polymeric ionic liquid enhanced all-solid-state electrolyte membrane for high-performance lithium-ion batteries. Electrochimica Acta.

[8] Costa L.T., Sun B., Jeschull F., Brandell D. (2015). Polymer-ionic liquid ternary systems for Li-battery electrolytes: Molecular dynamics studies of LiTFSI in a EMIm-TFSI and PEO blend. J. Chem. Phys..

[9] Shaplov A.S., Marcilla R., Mecerreyes D. (2015). Recent Advances in Innovative Polymer Electrolytes based on Poly(ionic liquid)s. Electrochimica Acta.

[10] Löwe R., Hanemann T., Hofmann A. (2019). Polymerizable Ionic Liquids for Solid-State Polymer Electrolytes. Molecule.

[11] Atkins P.W., de Paula J., Bär M. (2013). Physikalische Chemie.

[12] Tanner J.E. (1970). Use of the Stimulated Echo in NMR Diffusion Studies. J. Chem. Phys..

[13] Stejskal E.O., Tanner J.E. (1965). Spin Diffusion Measurements: Spin Echoes in the Presence of a Time-Dependent Field Gradient. J. Chem. Phys..

[14] Zhang H., Li L., Feng W., Zhou Z., Nie J. (2014). Polymeric ionic liquids based on ether functionalized ammoniums and perfluorinated sulfonimides. Polym..

[15] Ogihara W., Washiro S., Nakajima H., Ohno H. (2006). Effect of cation structure on the electrochemical and thermal properties of ion conductive polymers obtained from polymerizable ionic liquids. Electrochimica Acta.

[16] Ohno H., Yoshizawa M., Ogihara W. (2004). Development of new class of ion conductive polymers based on ionic liquids. Electrochimica Acta.

[17] Appetecchi G.B., Montanino M., Zane D., Carewska M., Alessandrini F., Passerini S. (2009). Effect of the alkyl group on the synthesis and the electrochemical properties of N-alkyl-N-methyl-pyrrolidinium bis(trifluoromethanesulfonyl)imide ionic liquids. Electrochimica Acta.

[18] Shin J.-H., Henderson W.A., Tizzani C., Passerini S., Jeong S.-S., Kim K.-W. (2006). Characterization of Solvent-Free Polymer Electrolytes Consisting of Ternary PEO–LiTFSI–PYR[sub 14] TFSI. J. Electrochem. Soc..

[19] Bruce P. (1988). Conductivity and transference number measurements on polymer electrolytes. Solid State Ionics.

[20] Xue Z., He D., Xie X. (2015). Poly(ethylene oxide)-based electrolytes for lithium-ion batteries. J. Mater. Chem. A.

[21] Stephan A.M. (2006). Review on gel polymer electrolytes for lithium batteries. Eur. Polym. J..

[22] Diddens D., Heuer A. (2014). Simulation Study of the Lithium Ion Transport Mechanism in Ternary Polymer Electrolytes: The Critical Role of the Segmental Mobility. J. Phys. Chem. B.

[23] Zhang Z., Nasrabadi A.T., Aryal D., Ganesan V. (2020). Mechanisms of Ion Transport in Lithium Salt-Doped Polymeric Ionic Liquid Electrolytes. Macromolecules.

[24] Long L., Wang S., Xiao M., Meng Y. (2016). Polymer electrolytes for lithium polymer batteries. J. Mater. Chem. A.

[25] Qian J., Jin B., Li Y., Zhan X., Hou Y., Zhang Q. (2021). Research progress on gel polymer electrolytes for lithium-sulfur batteries. J. Energy Chem..

