# Supplementary material for: Structure–Property Relationship of Polymerized Ionic Liquids for Solid-State Electrolyte Membranes
Source: Polymers (Basel). 2021 Mar 4;13(5):792. doi: 10.3390/polym13050792 (PMC7961940; doi:10.3390/polym13050792)
Supplement: Supplementary file 1 [file polymers-13-00792-s001.pdf]

polymers

Supplementary

Structure–Property Relationship of Polymerized Ionic Liquids for Solid-State Electrolyte Membranes

Robert Löwe [1,2], Thomas Hanemann [1,2], Tatiana Zinkevich [1] and Andreas Hofmann [1,*]

1 Institute for Applied Materials, Karlsruhe Institute of Technology, Hermann-von-Helmholtz-Platz 1, D-76344 Eggenstein-Leopoldshafen, Germany; robert.loewe@kit.edu (R.L.), thomas.hanemann@kit.edu (T.H.), tatiana.zinkevich@kit.edu (T.Z.)
2 Department of Microsystems Engineering, University of Freiburg, Georges-Köhler-Allee 102, D-79110 Freiburg, Germany
* Correspondence: andreas.hofmann2@kit.edu; Tel.: +49-721-608-25920

Citation: Löwe, R.; Hanemann, T.; Zinkevich, T.; Hofmann, A. Structure–Property Relationship of Polymerized Ionic Liquids for Solid-State Electrolyte Membranes. *Polymers* **2021**, *13*, 792. https://doi.org/10.3390/polym13050792

Received: 18 February 2021
Accepted: 3 March 2021
Published: 4 March 2021

Publisher's Note: MDPI stays neutral with regard to jurisdictional claims in published maps and institutional affiliations.

Copyright: © 2021 by the authors. Licensee MDPI, Basel, Switzerland. This article is an open access article distributed under the terms and conditions of the Creative Commons Attribution (CC BY) license (http://creativecommons.org/licenses/by/4.0/).

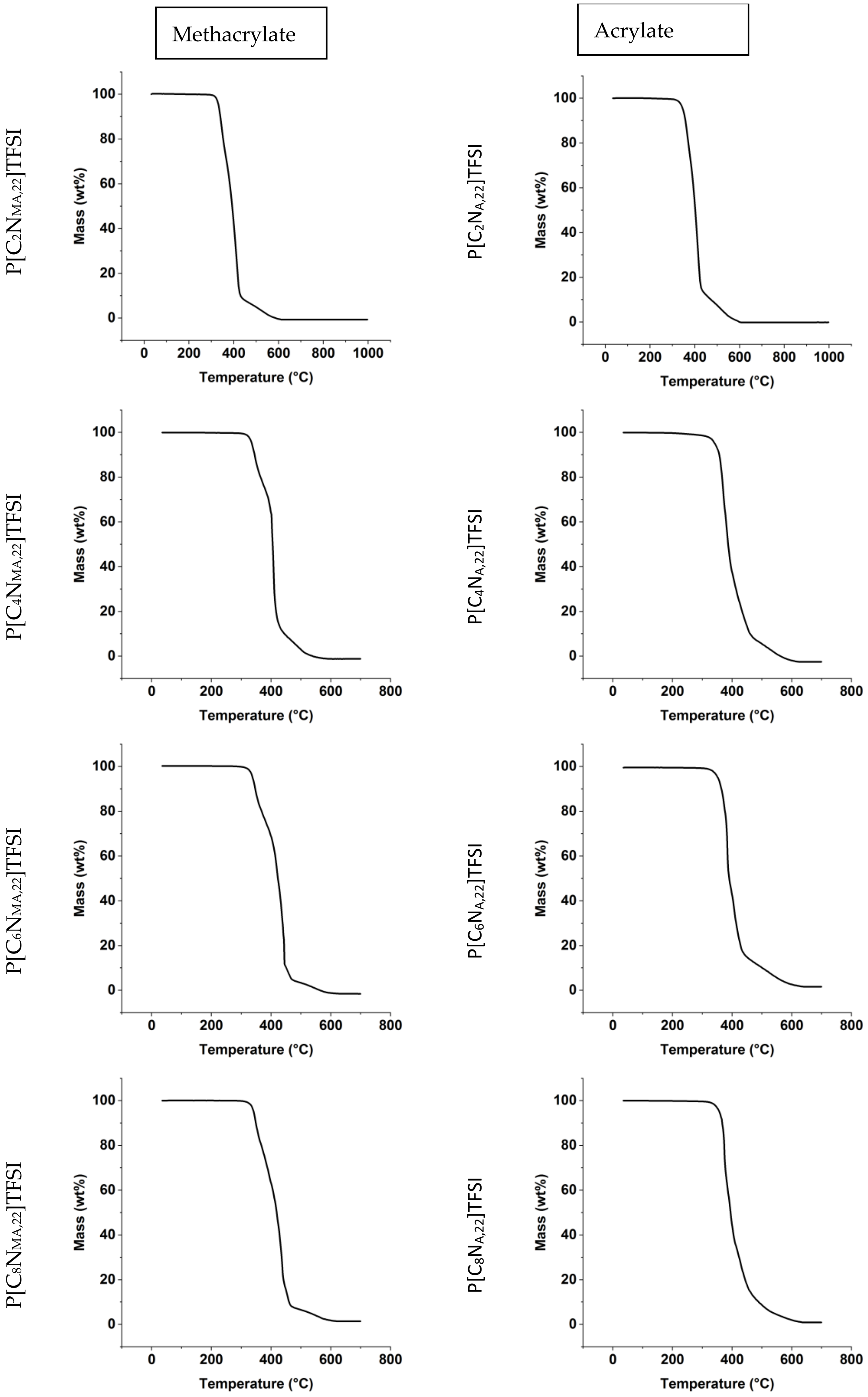

Figure S1. TGA curves of the eight studied PIL materials without addition of conducting salt.

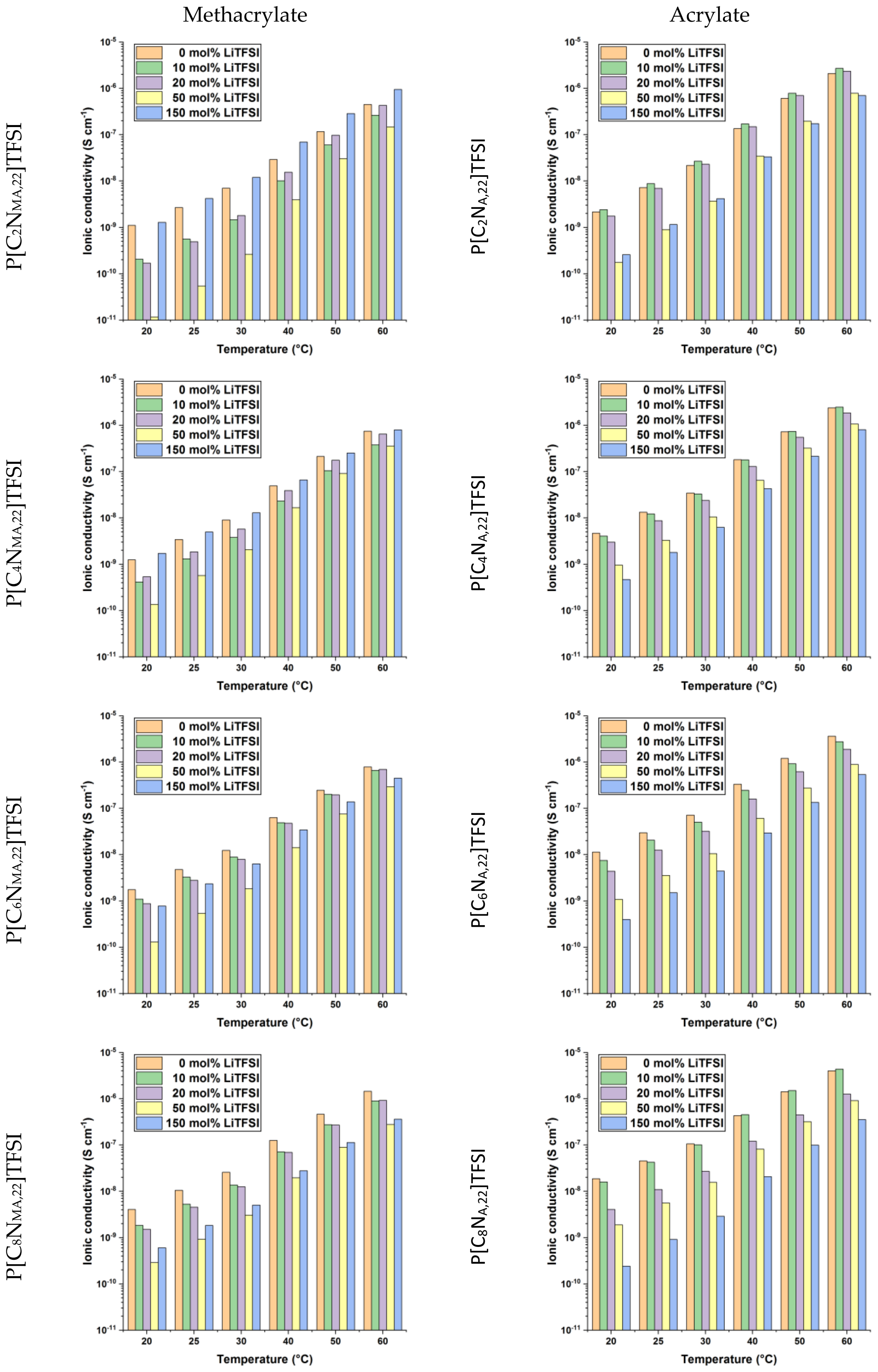

Figure S2. Ionic conductivity values of the membranes in dependence of monomer structure, temperature and LiTFSI concentration.
